# Synthesis and Application of Polypyrrole/Fe_3_O_4_ Nanosize Magnetic Adsorbent for Efficient Separation of Hg^2+^ from Aqueous Solution

**DOI:** 10.1002/gch2.201700078

**Published:** 2017-12-27

**Authors:** Zohreh Falahian, Firoozeh Torki, Hossein Faghihian

**Affiliations:** ^1^ Department of Chemistry Islamic Azad University Shahreza branch Shahreza 8648146411 Iran

**Keywords:** adsorption, core–shell, magnetic nano‐adsorbents, mercury, polypyrrole

## Abstract

In order to prepare the magnetic adsorbent, polymerization of pyrrole is performed in a mixture containing Fe_3_O_4_ and FeCl_3_. FTIR, XRD, SEM, EDAX, BET and VSM techniques are employed to characterize the synthesized adsorbent. The results indicate that a homogeneous film of polypyrrole is formed on the surface of magnetic material. The synthesized adsorbent uptakes 173.16 mg g^−1^ of Hg^2+^ from aqueous solution, which is superior to the previously reported results for a similar adsorbent. Magnetic performance of the adsorbent is sufficient to separate the used adsorbent from the solution by use of a magnetic bar placed outside of the vessel. Langmuir, Freundlich, Temkin, Redlich–Peterson, and Sips isotherm models are employed to evaluate the experimental adsorption data. The kinetic models are studied and the experimental data are described by the pseudo‐second‐order kinetic model. The calculated thermodynamic parameter shows that the sorption process is endothermic and spontaneous. Regeneration of the used adsorbent indicates that more than 90% of the initial capacity remains after regeneration.

## 1 Introduction

In recent years, water pollution caused by heavy metals has become one of the major economic and environmental problems and intensive attentions have been paid to the widely distribution of these cations into the environment.^1^ Mercury is one of the most toxic heavy metals. It accumulates in living tissues and causes many damages to the human body organism. Mercury has toxic effects on reproduction, the central nervous system, liver, and kidneys, and causes pulmonary dysfunction, chest pain, dyspnea, sensory, and psychological impairments.^1^

Many industrial processes such as coal combustion, natural‐gas scrubbing, metallurgy, oil refineries, rubber processing, power plants, paper, and fertilizer generate mercury becoming the main source of mercury in aquatic ecosystem and waste streams.^1^ Mercury is also used for the manufacture of industrial chemicals or for electrical and electronic applications. It is also used to produce chlorine gas, caustic soda, medical devises, dental fillings, and batteries.

The European Union, the US Environmental Protection Agency (EPA) and the World Health Organization (WHO) have put restriction of 1 µg L^−1^ of mercury in water and 5 µg L^−1^ for wastes discharged into the environment.^2^ Therefore, the residual mercury concentration of industrial wastewater and drinking water must be brought below the safety limit.

The convention methods used to treat mercury containing aqueous solutions include precipitation, extraction, reverse osmosis, membrane technologies, ion exchange, and adsorption.^3^ Because of simplicity, convenience, and high efficiency, adsorption process is one of the most popular methods and attracted considerable attentions for removal of heavy metal cations.^4^ Application of magnetic adsorbents for removal of target pollution is deeply studied. However, nanosized magnetic particles can be oxidized in air and easily aggregated in aqueous systems. To avoid the drawbacks of nanosized magnetic particles, preparation of core–shell structure has been suggested.^5^ Polypyrrole which is a low cost and degradable polymer with high chemical resistant is a suitable candidate for preparation of core–shell adsorbents.^6^

In this research, a core–shell magnetic nano adsorbent was synthesized by combination of polypyrrole and Fe_3_O_4_ (PPy/Fe_3_O_4_). The nano‐sized adsorbent was employed to uptake Hg^2+^ from aqueous solutions.

## 2 Results and Discussion

### 2.1 Nanosized PPy/Fe_3_O_4_ Characterization

The Fourier transform infrared (FTIR) spectra of Fe_3_O_4_ (Figure S1a, Supporting Information), showed an absorption band at 3386 cm^−1^ assigned to the stretching vibration of O—H.^5^ The bands at 461 and 571 cm^−1^ corresponded to of Fe—O vibrational stretching.^7^, ^8^ In the FTIR of PPy/Fe_3_O_4_ (Figure S1b, Supporting Information), the bands at 3636 and 3311 cm^−1^ assigned respectively to N—H vibration stretching of PPy and O—H^9^ and adsorption band at 1545 cm^−1^ belonged to N—H amine of pyrrole ring.^10^ The peaks observed at 1476, 1422, 1034, 788, and 849–916 cm^−1^ attributed, respectively to ring stretching of pyrrole, conjugated C—N, stretching of C—N, C—H vibrational stretching and C—H deformation.^6^, ^11^ Bands observed at 1165 and 1794 cm^−1^ are assigned to N—H in‐plane deformation and C—C, C=C stretching vibration.^12^ The presence of these bands indicated that polypyrrole was grafted on the surface of the nano adsorbent.

**Figure 1:**
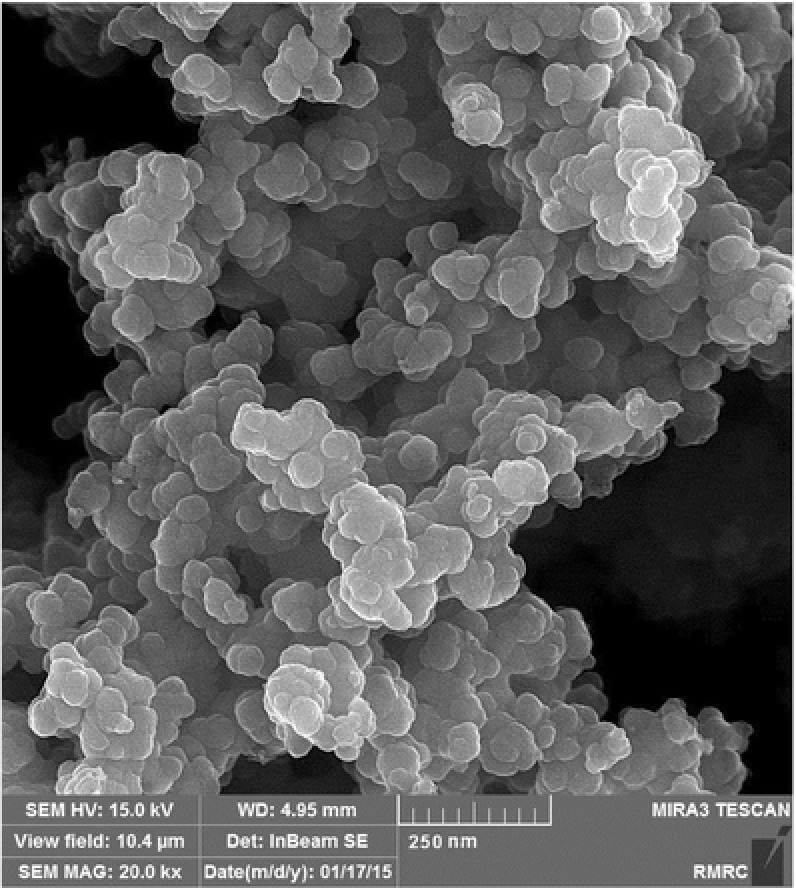
SEM image of PPy/Fe_3_O_4_.

**Figure 2:**
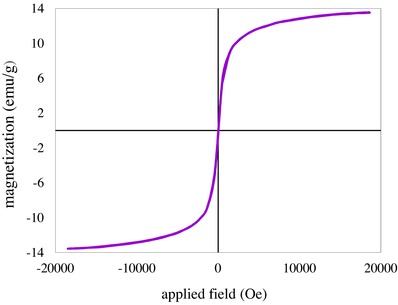
Magnetization curve of the PPy/Fe_3_O_4_ at room temperature.

Nanocomposite X‐ray diffractometer (XRD) pattern (Figure S2, Supporting Information) showed several diffraction lines appeared at 2θ = 30.00, 40.00, 35.40, 42.86, 53.34, 57.16 and 62.86° are the characteristic peaks of Fe_3_O_4_. The board diffraction line observed at 2θ = 25 was the characteristic peak of PPy.^10^ The XRD pattern indicated that the crystal structure of Fe_3_O_4_ remained intact during the polymerization process. The surface area of the nanosized adsorbent was determined by Brunauer–Emmett–Teller (BET) technique (Figure S3, Supporting Information). The distance between adsorption and desorption plots was negligible indicating that the pore sizes of the adsorbent remained unchanged during the processes. The low specific surface area of the adsorbent indicated that the surface of the magnetic core was homogenously covered by a film of polymer and the magnetic core–shell was produced. In the scanning electron microscopy (SEM) image of the adsorbent, the aggregation of the uniform spherical beads was clearly observed (**Figure** **1**). The average particle size of 50 nm was estimated. Surface elemental analysis of the adsorbent studied by energy dispersive X‐ray spectroscopy (EDAX) (Figure S4, Supporting Information) showed that N, O, C, and Fe elements were presented on the surface of the adsorbent. The low intensity of the Fe and O lines indicated that the magnetic Fe_3_O_4_ core was properly covered by polypyrrole. The most important feature of the synthesized adsorbent was its magnetic property, studied by vibrating sample magnetometer (VSM) method (**Figure** **2**). The magnetic curve without hysteresis loop and remanence was obtained indicating the suitable super paramagnetic property of the synthesized PPy/Fe_3_O_4_. The low saturation magnetization of 13.55 emu g^−1^ exhibited by the sample indicated that the polymer was uniformly and properly shielded the magnetic core of the adsorbent. In practice, the magnetization of the adsorbent was sufficient to separate it from solution by an external permanent magnet. The remanence of zero ensured that the sample lost its magnetization after removing of magnetic field.

### 2.2 Adsorption Experiments

#### 2.2.1 Influence of Concentration

**Figure 3:**
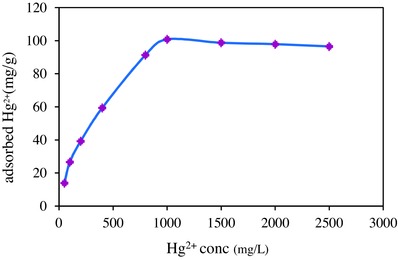
Effect of initial concentration of Hg^2+^ on adsorption capacity.

To study the influence of concentration on the removal efficiency of Hg^2+^, the removal efficiency was measured in concentration range of 50–2500 mg L^−1^ (**Figure** **3**). By increasing of concentration, first significant increase on the adsorption capacity was observed, and then at concentration higher than1000 mg L^−1^ the trend was almost constant. By increasing initial concentration, the interaction between Hg^2+^ and adsorption sites was facilitated because diffusion of cations to the adsorption sites proceeded more quickly. When the adsorption sites were fully engaged, the equilibration was established and constant adsorption capacity was observed. Similar results were reported for the adsorbent prepared by magnetite particles coated by silica,^8^ polypyrrole/Fe_3_O_4_ nanosized magnetic adsorbent used for removal of fluoride from aqueous solution^11^ and polypyrrole composites used for separation of mercury from solutions.^13^ The selectivity of the synthesized adsorbent was measured in the presence of Cd^2+^ ions. The Hg^2+^ concentration was kept at 500 mg L^−1^ while the Cd^2+^ concentration was gradually increased from 50 to 2000 mg L^−1^. The tolerance limit was defined as the highest concentration of coexisting cation, which changes the recovery of chromate less than ±5% of the initial value. It was concluded that at concentration below of 100 mg L^−1^ of Cd^2+^, the adsorbent was selective toward Hg^2+^.

#### 2.2.2 Effect of pH

**Figure 4:**
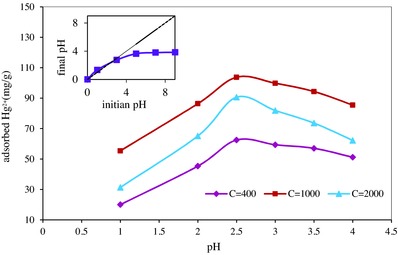
Effect of pH on Hg^2+^ removal, pH_PZC_ of the adsorbent.

The solution pH strongly affected the removal efficiency of mercury (**Figure** **4**). At lower pH, because of protonation, the adsorbent surface was positively charged, and repulsion electrostatic forces restricted sorption of Hg^2+^. Additionally, at this condition, H_3_O^+^ ions competed with Hg^2+^ for the sorption sites and limited the adsorption capacity for mercury.^14^ At high pH, according to the following reactions, soluble $\mathrm{Hg}{\left(\mathrm{OH}\right)}_{3}^{-}$ and $\mathrm{Hg}{\left(\mathrm{OH}\right)}_{4}^{-}$ ions were formed which are less adsorbed by the adsorbent 15

(1) $${\mathrm{Hg}}^{2+}+2{\mathrm{OH}}^{-}\to \mathrm{Hg}{\left(\mathrm{OH}\right)}_{2\;}\;K_{\mathrm{sp}}=3.6\times 10^{-26}$$

(2) $$\mathrm{Hg}{\left(\mathrm{OH}\right)}_{2}+\mathrm{OH}\to \mathrm{Hg}{\left(\mathrm{OH}\right)}_{3}^{-}\;\log\;K_{1}=10.6$$

(3) $$\mathrm{Hg}{\left(\mathrm{OH}\right)}_{3}^{-}+{\mathrm{OH}}^{-}\to \mathrm{Hg}{\left(\mathrm{OH}\right)}_{4}^{2-}\;\log\;K_{2}=11.2$$

The measured pH_PZC_ of the nanocomposite was 2.4 indicating that at pH < 2.4, the surface charge of the adsorbent became positive and repulsion force limited the adsorption of mercury.^16^ (Figure 4). At pH higher than pH_PZC_, the surface became negative and Hg^2+^ ions were more attracted to the adsorption sites.^17^ Therefore, the optimized capacity obtained at pH = 2.5, slightly higher than pH_PZC_. In a Similar study, polypyrrole‐reduced graphene oxide composite was evaluated for removal of mercury and the optimized capacity was obtained at pH = 5.^18^

#### 2.2.3 Influence of Contacting Time

**Figure 5:**
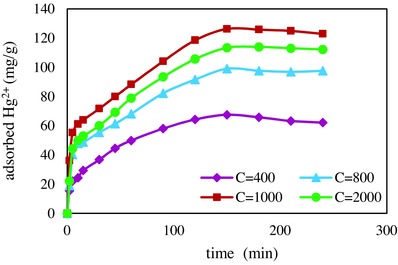
Effect of contact time on Hg^2+^ removal.

As shown in (**Figure** **5**), the sorption process was initially very fast and then slowed until equilibration was established within 140 min. More than 50% of the adsorption capacity obtained within 10 min and the remaining capacity achieved within 140 min. Chandra et al. used polypyrrole‐reduced graphene oxide for adsorption of mercury and reported that the optimized contacted time was 180 min.^18^

#### 2.2.4 Influence of Temperature

**Figure 6:**
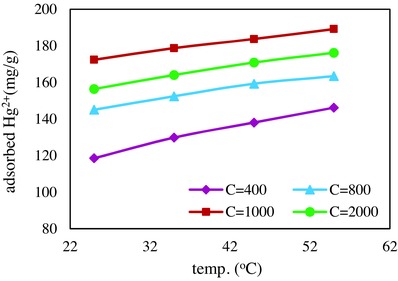
Effect of temperature on the adsorption of Hg^2+^.

Removal of Hg^2+^ by nano adsorbent was studied at four different temperatures from 298 to 328 K (**Figure** **6**). The increase in the adsorption capacity which occurred by increasing of temperature was an indication of endothermic nature of the process. Thermodynamic parameters of the adsorption process were estimated by the data obtained from this experiment. ∆*H* ^o^ and ∆*S* ^o^ were calculated from the slopes and the intercepts of the linear plot of the following equation

(4) $$ln\left(K_{d}\right)=\;\frac{\Delta S^{0}}{R}-\frac{\Delta H^{0}}{RT}$$

Where Δ*H*° is the change in enthalpy (J mol^−1^) and Δ*S*° (J mol^−1^ K^−1^) is the entropy changes. *K*_d_ is the distribution coefficient, *R* is the universal gas constant (8.314 J mol^−1^ K^−1^), and *T* is the absolute temperature (K). The values of enthalpy change (Δ*H*°) and entropy change (Δ*S*°) were calculated from the slope and intercept of the line obtained by plotting ln(*K*_d_) versus (1/*T*) (Figure S5, Supporting Information).^19^ ∆*G* ^o^ was calculated by the following equation and the data are given in Table S1 in the Supporting Information

(5) $$\Delta G^{0}=\;\Delta H^{0}-T\Delta S^{0}$$

The negative value for ∆*G* ^o^ was the indication of spontaneous nature of the process. Positive value of ∆*S* ^o^ suggested that upon mercury adsorption, disorder increased at the solid–liquid interface and the positive ∆*H* ^o^ showed that the adsorption process was endothermic.

### 2.3 Study of Adsorption Isotherms

Adsorption isotherms were constructed by plotting of the amount of adsorbed mercury by unit mass of the adsorbent versus the concentration of ions in the solution. In this study, isotherm models; Langmuir and Freundlich and Temkin, Redlich–Peterson and Sips were used to describe the Hg^2+^ adsorption by the magnetic adsorbent.

#### 2.3.1 The Langmuir Isotherm

Langmuir isotherm^20^ describes the adsorption of cations on the surface of sorbent from a liquid solution as linear and nonlinear forms given in Equations (6) and (7)

(6) $$q_{e}=\frac{q_{m}K_{L}C_{e}}{1+K_{L}C_{e}}$$

(7) $$\frac{C_{e}}{q_{e}}=\frac{1}{q_{m}K_{L}}+C_{e}\left(\frac{1}{q_{m}}\right)$$

*C*_e_, the equilibrium concentration (mg L^−1^), *q*_e,_ equilibrium adsorption capacity (mg g^−1^), *q*_m_ (mg g^−1^), and *k*_L_ (L mg^−1^) are respectively the capacity and binding energy of adsorption. Plot of *C*_e_/*q*_e_ versus *C*_e_ gave a straight line of slope 1/*q*_m_ and an intercept of 1/(*q*_m_ *K*_L_) Figure S6a in the Supporting Information.

The Langmuir isotherm indicated that the surface of adsorbent was homogeneous and the adsorption was monolayer with uniform energy. The essential characteristics of the Langmuir isotherm can be expressed in terms of a dimensionless constant separation factor *R*_L_ 21 given by Equation (9)

(8) $$R_{L}=\frac{1}{1+K_{L}C_{0}}$$

Where *C*_0_ (mg L^−1^) is the highest initial concentration of adsorbent and *K*_L_ (L mg^−1^) is Langmuir constant. The *R*_L_ indicates the nature of the isotherm accordingly: 0 < *R*_L_ < 1 shows favorable adsorption and RL = 0 indicates irreversible adsorption.

The value of *R*_L_ in the present study was found to be 0.25, 0.09, 0.14, and 0.12 at 298, 308, 318, and 325 K, respectively indicating that the adsorption of Hg^+2^ on Fe_3_O_4_/PPy was favorable at the studied temperatures.

#### 2.3.2 The Freundlich Isotherm

The Freundlich isotherm,^22^ indicates that the surface was heterogeneous and distribution of adsorption heat is nonuniform. The linear and nonlinear form of Freundlich equation expressed in Equations (9) and (10)

(9) $$q_{m}=K_{F}C_{e}^{\frac{1}{n}}$$

(10) $$\log q_{e}\;=\;\log\;K_{F}+\frac{1}{n}\log C_{e}$$

*C*_e_ is the equilibrium concentration of ingoing cation (mg L^−1^), *q*_e_ is the equilibrium capacity (mg g^−1^). *K*_F_ and *n* are Freundlich constants related to the adsorption capacity and adsorption intensity, respectively, were obtained from intercept and slope of straight line obtained by plotting log *q*_e_ versus log *C*_e_ (Figure S6b, Supporting Information).

### 2.3.3 The Temkin Isotherm

Based on Temkin isotherm^23^ the adsorbent surface is heterogeneous, binding energy is distributed uniformly and heat of adsorption decreases linearly with adsorption quantity. The linear and nonlinear forms of Temkin isotherm express as Equations (11) and (12) as follows

(11) $$q_{e}=\frac{RT\;\ln\;\left(A_{T}C_{e}\right)}{b_{T}}$$

(12) $$q_{e}=B_{T}\ln\;\left(K_{T}\right)+B_{T}\ln\;\left(C_{e}\right)$$

*C*_e_, equilibrium concentration (mg L^−1^) and *q*_e_ equilibrium adsorption capacity (mg g^−1^), *A*_T_ (L mg^−1^) and *b*_T_ (J.mg^−1^) are Temkin constant and *R* is the universal gas constant. By plotting *q*_e_ versus ln(*C*_e_), the constants parameters were obtained from slope and intercept of the straight‐line (Figure S6c, Supporting Information).

#### 2.3.4 The Redlich–Peterson Isotherm

The Redlich–Peterson isotherm^24^ contains three parameters and involves the features of both the Langmuir and the Freundlich isotherms. It can be described by following equations

(13) $$q_{e}=\frac{AC_{e}^{}}{1+BC_{e}^{g}}$$

(14) $$\ln\left(A\;\frac{C_{e}}{q_{e}}-1\right)=g\ln\left(C_{e}\right)+\ln\left(B\right)$$

The isotherm constants, *A*, *B*, and *g*, were evaluated by the pseudo‐linear plot represented in Equation (14) by using a trial‐and‐error procedure to determine the coefficient of determination, *R* ^2^ for a series of values of *A* for the linear regression of ln[*A*(*C*_e_/*q*_e_) − 1] versus ln(*C*_e_) and to obtain the best value of *A* which yields a maximum value of *R* ^2^ (Figure S6d, Supporting Information). When *g* is equal to 1, that isotherm reduced to Langmuir model and showed the homogeneity of the adsorbent surface. When *A* is equal to zero, it is the same as the Freundlich isotherm equation. *B* is a measure of adsorption affinity.^25^

#### 2.3.5 The Sips Isotherm

The Sips isotherm is a combination of Langmuir and Freundlich models predicting nonuniform surfaces on adsorption system.^26^ Mathematically, it can be described as follows

(15) $$q_{e}=\frac{q_{\mathrm{ms}}K_{s}C_{e}^{m}}{1+k_{s}C_{e}^{m}}$$

The linear form of Sips equation is as follows

(16) $$\frac{1}{q_{e}}=\frac{1}{Q_{\max}K_{s}}{\left(\frac{1}{C_{e}}\right)}^{1/m}+\left(\frac{1}{Q_{\max}}\right)$$

Where *Q*_max_ is indicator of Sips maximum adsorption capacity (mg g^−1^), *K*_S_, and *m* are the Sips equilibrium constant (L mg^−1^) and model exponent respectively. By plotting 1/*q*_e_ versus (1/*C*_e_)^1/^ *^m^* in the pseudo‐linear plot of Equation (17), a straight line was obtained (Figure S6e, Supporting Information). The Sips isotherm equation is characterized by the dimensionless heterogeneity factor, *m*, which can also be employed to describe the system's heterogeneity when m is between 0 and 1. When *m* = 1, the Sips equation reduces to the Langmuir equation and it implies a homogeneous adsorption process.^27^

### 2.4 The Error Analysis

In order to estimate the best fitted model for the adsorption system, it was necessary to analyze the data using error analysis. The calculated expressions of some error functions; the sum of the squares of the errors (SSE), the average relative errors (ARE), and chi square are given in Equations (17)–(19) as follows

(17) $$\mathrm{SSE}=\sum {\left(q_{c}-q_{e}\right)}^{2}$$

(18) $$\mathrm{ARE}=\frac{\sum \left|\left(q_{c}-q_{e}\right)/q_{e}\right|}{n}$$

(19) $${ℵ}^{2}=\;\sum \;\frac{{\left(q_{e}-q_{c}\right)}^{2}}{q_{e}}$$

**Figure 7:**
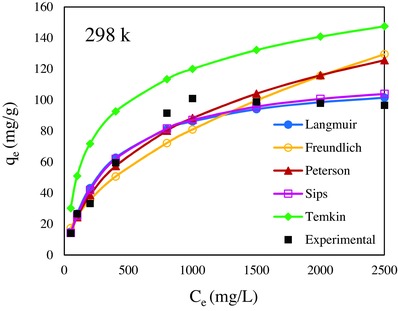
The nonlinear plot of Langmuir, Freundlich, Temkin, Redlich–Peterson, Sip adsorption isotherms, and experimental data at 298 K.

*q*_c_ and *q*_e_ are, respectively, the calculated adsorption capacity and the experimental adsorption capacity at equilibrium (mg g^−1^) and *n* is number of experimental points data. The nonlinear isotherm plots of the experimental data, Langmuir, Freundlich, Temkin, Redlich–Peterson, and Sips isotherms at 298 is shown in **Figure** **7** and 308, 318, and 325 K are shown in Figure S7a–c, respectively.

According to results obtained for the studied isotherms, the linear analysis the *R* ^2^ values less affected. Therefore, the nonlinear chi square, ARE, and SSE analysis might be methods of avoiding such errors. The error analysis values of models indicated that Sips isotherm is best‐fitted model to the experimental data. The Freundlich and Temkin isotherms exhibited the highest chi square, ARE and SSE values than the other isotherms which was not considered to be fit model. The *m* parameter of Sips model is other justification of homogeneity of adsorbent surface that at all temperature *m* is equal to 1. But in 318 K, *m* is close to 1 and confirmed which most of surface is homogenized.

The values obtained for constant parameters of the adsorption isotherms and error analysis is given in Tables S2 and S3 in the Supporting Information.

### 2.5 Kinetic Studies

The kinetic of adsorption process studied by Pseudo‐first order, Pseudo‐second order, Intraparticle diffusion, and Elovich kinetic models. In the present work, a kinetic analysis approach to the sorption process was adopted and various sorption parameters such as the equilibrium sorption capacity, rate constants, and initial sorption rate were calculated from the experimental data by suitable kinetic model.

#### 2.5.1 The Pseudo‐First Order Kinetic Model

Linear and nonlinear form of kinetic model; pseudo‐first order as expressed in Equations (20) and (21) were used^28^

(20) $$\log\left(q_{e}-\;q_{t}\right)=\log q_{e}-\left(\frac{K_{1}}{2.303}\right)t$$

*K*_1_ is the pseudo‐first order rate constant (min^−1^) of adsorption and *q*_e_ and *q_t_* are the adsorption capacities of Hg^2+^ (mg g^−1^) at equilibrium and at time *t* (min) respectively. The kinetic parameters were calculated from the slope and intercept of the linear plots of log(*q*_e_ – *q_t_*) versus *t* (Figure S8, Supporting Information)^29^

(21) $$q_{t}=\;q_{e}\;\left[1-\;e^{-Kt}\right]$$

#### 2.5.2 The Pseudo‐Second Order Kinetic Model

The linear and nonlinear forms of pseudo second order equation are as given in Equations (22) and (23)

(22) $$\frac{t\;}{q_{t}}=\frac{1}{K_{2}q_{e}^{2}}+\left(\frac{1}{q_{e}}\right)t$$

(23) $$q_{t}=\;\frac{tK_{2}q_{e}^{2}}{1+tK_{2}q_{e}}$$

*K*_2_ is the pseudo‐second orders rate constant of adsorption (g mg^−1^ min^−1^). *q*_e_ and *q_t_* are the adsorption capacities (mg g^−1^) at equilibrium and at time *t* (min), respectively. The constant parameters of model was identified from the slope and intercept of the pseudo‐linear plot of *t*/*q_t_* versus *t* (Figure S9, Supporting Information)

#### 2.5.3 The Intraparticle Diffusion Kinetic Model

The Intraparticle diffusion model expressed as the following equation

(24) $$q_{t}=K_{i}t^{\frac{1}{2}}+C_{i}$$

Where *K*_i_ is the diffusion rate constant (mg g^−1^ min^−1/2^) and *C*_i_ is intraparticle diffusion constant intercept of the line (mg g^−1^). It is directly proportional to the boundary layer thickness. By plotting *q*_e_ versus ln(*t* ^1/2^), the constant parameters were obtained from the slope and intercept of the obtained straight line, respectively. If the regression of *q_t_* versus *t* ^1/2^ is linear and passes through the origin, then intraparticle diffusion is rate‐limiting step.^30^ But deviation of the line from the origin further shows that intraparticle transport is not the only rate limiting step. Probably the transport of the sorbent through the particle sample interphase onto the pores of the particles, as well as adsorption on the available surface of the adsorbent, is responsible for the adsorption.^31^ For intraparticle diffusion plots, the first, sharper region is the immediately adsorption or external surface adsorption. The second region is the gradual adsorption stage where intraparticle diffusion is the rate limiting. In some cases, the third region exists, which is the final equilibrium stage where intraparticle diffusion starts to slow down due to the extremely low adsorbate concentrations left in the solutions.^32^ As seen from Figure S10 in the Supporting Information, the plot was not linear over the whole time range, implying that more than one process affected the adsorption.

#### 2.5.4 The Elovich Kinetic Model

The linear and nonlinear forms of Elovich kinetic model are explained as Equations (25) and (26) as follows

(25) $$q_{t}=\frac{\ln\left(\alpha \beta \right)}{\beta }+\frac{1}{\beta }\ln\left(t\right)$$

(26) $$q_{t}=\frac{1}{\beta }\ln\left(\alpha \beta t+1\right)$$

*q_t_* is the sorption capacity at time *t* (mg g^−1^), α (mg g^−1^ min)^−1^, and β (g mg^−1^). By the plot of *q_t_* against ln(*t*), α  and β were respectively obtained from intercept and slop of straight line (Figure S11, Supporting Information). The nonlinear regression analysis, chi square, ARE, and SSE were showed better fitted kinetic model for adsorption of mercury from aqueous solutions. The summarized of kinetic parameters and results of error analysis are given in Table S4 in the Supporting Information.

**Figure 8:**
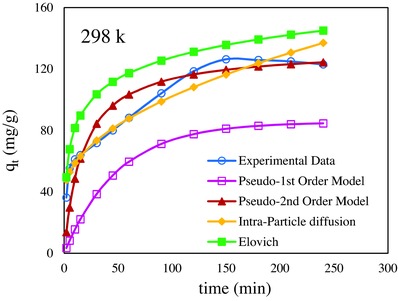
The nonlinear plot of Pseudo‐1st, Pseudo‐2nd, Intraparticle diffusion, Elovich kinetic models, and experimental data at 298 K.

According to the results, although, the correlation coefficients for models are close, but chi square, ARE, and SSE statistics are slightly better fitted experimental data to kinetic model. According to results obtained of analysis error, the second‐order kinetic model had lower chi square, ARE, and SSE and was more significant kinetic model to justified data.^33^ The second‐order kinetic model indicated that the adsorption process was chemical in nature and the rate‐limiting step of the adsorption was chemisorption without involvement of a mass transfer in solution.^34^ The nonlinear plot of *q_t_* against *t* was plotted to compare the fitness of the kinetic model to the experimental data (**Figure** **8**). It was concluded that that the experimental data were in good agreement with Pseudo‐second order kinetic model.

### 2.6 Reusability of the Adsorbent

Regeneration ability of adsorbents is an important factor to develop cost effective removal process. For regeneration study, the stability of the adsorbent against regeneration reagents is to be evaluated. The chemical stability of the core–shell adsorbent of this work was examined by putting 0.1 g of the adsorbent in contact with 50 mL of 1 m HNO_3_ solution for 24 h. The adsorbent was then separated by employing external magnetic field and the concentration of iron was measured in the solution. The Fe content in the remaining solution was negligible indicating that the adsorbent was stable in acidic solution.

To regenerate of the nano adsorbent, 0.2 g of the used adsorbent was shaken with 20 mL of 1.0 m HNO_3_ for 120 min and the regenerated adsorbent was separated by putting a magnet bar outside of the vessel. The adsorption–regeneration process was repeated for five times. It was concluded that adsorbent retained 90% of its initial capacity after first regeneration cycle (Figure S12, Supporting Information).

## 3 Conclusion

Nano adsorbent; polypyrrole/Fe_3_O_4_ was successfully prepared by in situ polymerization method. The characterization method performed by FTIR, XRD, SEM, EDAX, and BET techniques indicated that the core–shell adsorbent was synthesized. It was also revealed that the particles of the adsorbent were nanosized. VSM measurement indicated that the magnetic property of the nano adsorbent was sufficient for separation of the used adsorbent by employing an appropriate external magnetic field. The regeneration test showed that the adsorbent was chemically stable in acidic solutions. This was very beneficial for separation of mercury form waste streams which are mainly at acidic conditions. Under optimized conditions, the adsorbent showed high adsorption capacity of 173.16 mg g^−1^ which was excellent compared to the similar adsorbents. Adsorption process was kinetically fast, 50% of the capacity obtained within 10 min. The kinetic experimental data are described by the pseudo‐second‐order rate model. Adsorption data fitted to Sips isotherm which indicated homogeneous adsorption of Hg^2+^. Thermodynamic parameters indicated that the process was spontaneous and endothermic. The regeneration experiments showed that the adsorbent retained most of initial capacity.

## 4 Experimental Section

All chemical reagents purchased from Merck Company (Germany). Magnetic particles prepared by FeCl_3_.6H_2_O, FeCl_2_.4H_2_O, NaOH, and HCl. Pyrrole, acetone, and FeCl_3_ were used for synthesis of polypyrrole. Mercury supplied as mercury nitrate. XRD pattern were prepared by use of a Bruker, D8 Advanced XRD. FTIR spectra were taken by IR Spectrometer 65 instrument. SEM images were provided by Zeiss Ultra 55 instrument. The surface area, pore volume, and pore size of the samples determined by use of BET method (Quantochrome Nova2000, USA). Sample magnetization was measured by use of a Lake Shore7200 magnetometer.

*Preparation of Fe_3_O_4_*: Nanosized magnetic Fe_3_O_4_ was synthesized by coprecipitation technique. Briefly, 0.04 mole of ferric chloride and 0.02 mole of ferrous chloride were dissolved in 50 mL of 0.5 m HCl solution. The mixture was drop wise transferred to 500 mL of NaOH solution (1.5 m) at 80 °C under N_2_ atmosphere. The Fe_3_O_4_ nanoparticles were formed and separated from the solution. The particles were repeatedly rinsed with water and then dried at 50 °C.^35^

*Synthesis of PPy/Fe_3_O_4_*: PPY/Fe_3_O_4_ magnetic nano adsorbent was prepared through in situ polymerization method according to the modified procedure described by Bhaumik. A mixture prepared by dissolution of 0.4 g of Fe_3_O_4_ in 80 mL of deionized water was properly homogenized by ultrasonic method and after adding 6.0 g of FeCl_3_, the mixture was shaken for 10 min. 0.8 mL of pyrrole was added to the mixture and it was agitated for 3 h. The reaction was completed by adding a few mililiter of acetone to the reaction mixture. The nano adsorbent was separated and washed repeatedly with deionized water. The nano adsorbent was rinsed with acetone and dried at 100 °C for 6 h.^11^

*Adsorption Studies*: Mercury standard solutions (2–50 mg L^−1^) were prepared by the stock solution obtained by dissolution of accurately weighed amount of Hg(NO_3_)_2_ in deionized water. Adsorption experiments were conducted by adding 0.05 g of PPy/Fe_3_O_4_ into 15 mL of Hg^2+^ solution. After equilibration, by use a magnetic bar placed outside of the vessel, the used adsorbent was removed. The mercury concentration was measured in the remaining solution and the adsorption capacity was estimated by the Equation (27)

(27) $$q_{e}=\;\frac{\left(C_{i}-C_{e}\right)V}{W}$$

*q*_e_ is the adsorption capacity (mg g^−1^), *C*_i_ and *C*_e_ (mg L^−1^) are respectively initial and equilibrated concentration of mercury, *V* volume of the solution (L) and *W* (g) weight of dried PPy/Fe_3_O_4_. The influence of initial mercury concentration (50–2500 mg L^−1^), pH (1.00–400), contact time (2–250 min), and temperature (25–55 °C) on the adsorption capacity was measured by the same procedure.

## Conflict of Interest

The authors declare no conflict of interest.

## Supporting information

Supplementary
