# Supplementary material for: Synthesis and Application of Polypyrrole/Fe3O4 Nanosize Magnetic Adsorbent for Efficient Separation of Hg2+ from Aqueous Solution
Source: Glob Chall. 2017 Dec 27;2(1):1700078. doi: 10.1002/gch2.201700078 (PMC6607352; doi:10.1002/gch2.201700078)
Supplement: Supplementary file 1 — Supplementary [file GCH2-2-1700078-s001.pdf]

Copyright WILEY-VCH Verlag GmbH & Co. KGaA, 69469 Weinheim, Germany, 2017.

Global
Challenges
Open Access

Supporting Information

for *Global Challenges,* DOI: 10.1002/gch2.201700078

Synthesis and Application of Polypyrrole/Fe_3O_4 Nanosize Magnetic Adsorbent for Efficient Separation of Hg^{2+} from Aqueous Solution

*Zohreh Falahian, Firoozeh Torki, and Hossein Faghihian**

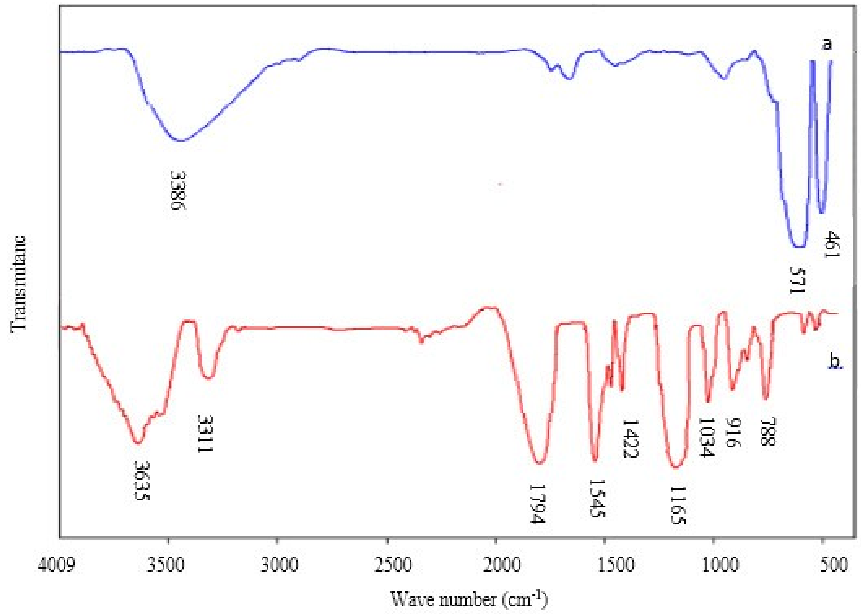

Fig.1S. FT-IR spectrum of PPy/Fe_3O_4

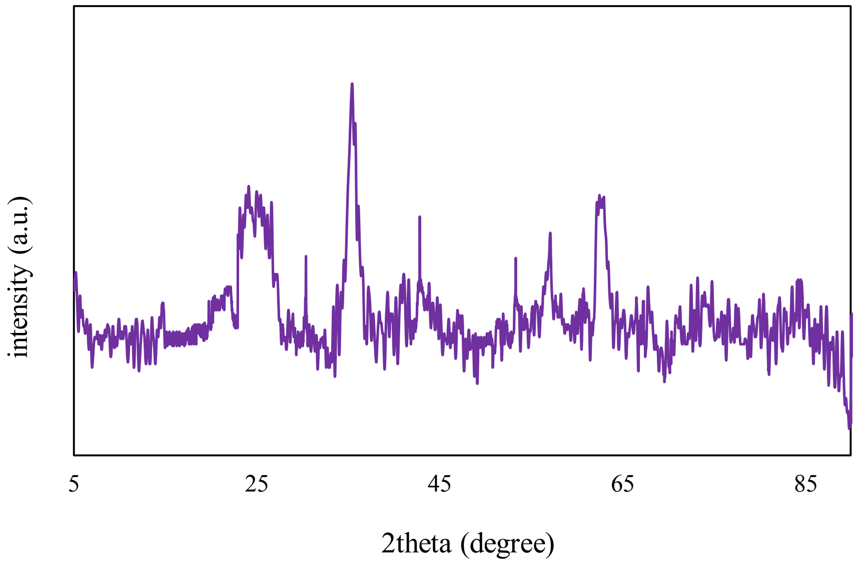

Fig. 2S. XRD pattern of PPy/Fe_3O_4

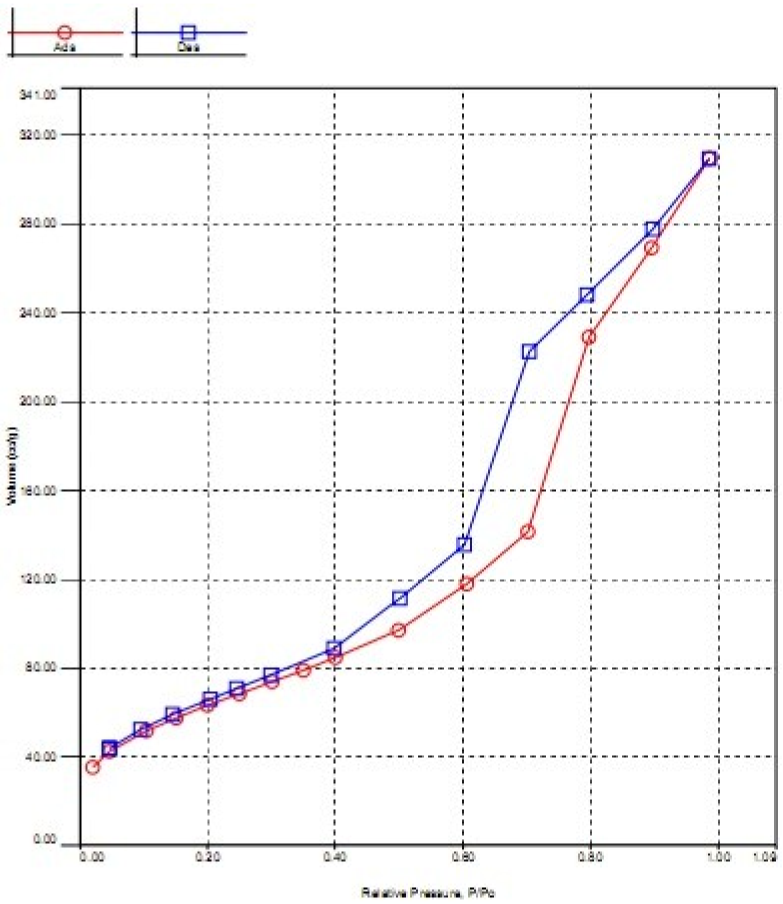

Fig. 3S. BET graph of PPy/Fe_3O_4

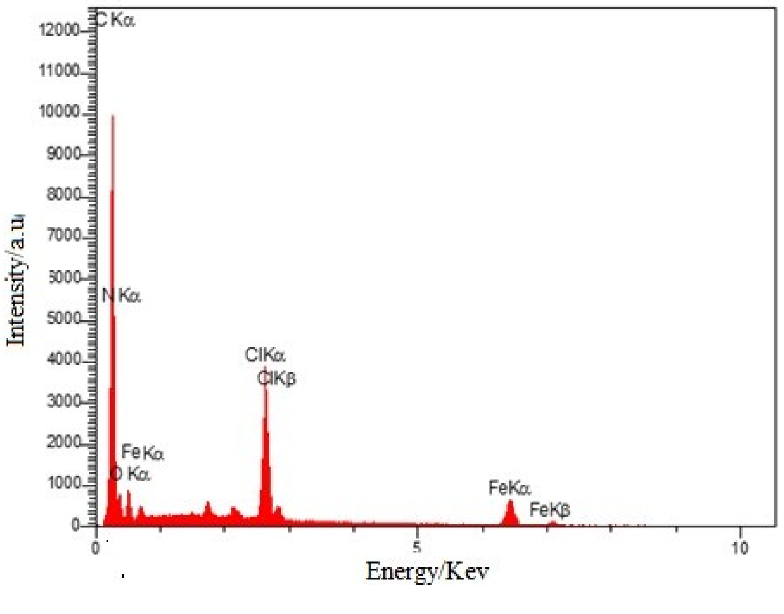

Fig. 4S. EDAX spectrum of PPy/Fe_3O_4

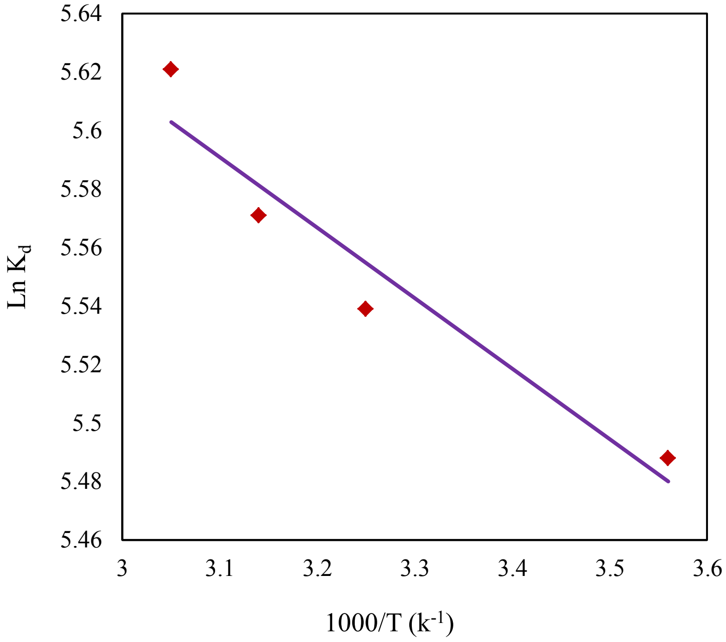

Fig. 5S. Plot of Ln (k_d) versus 1/T

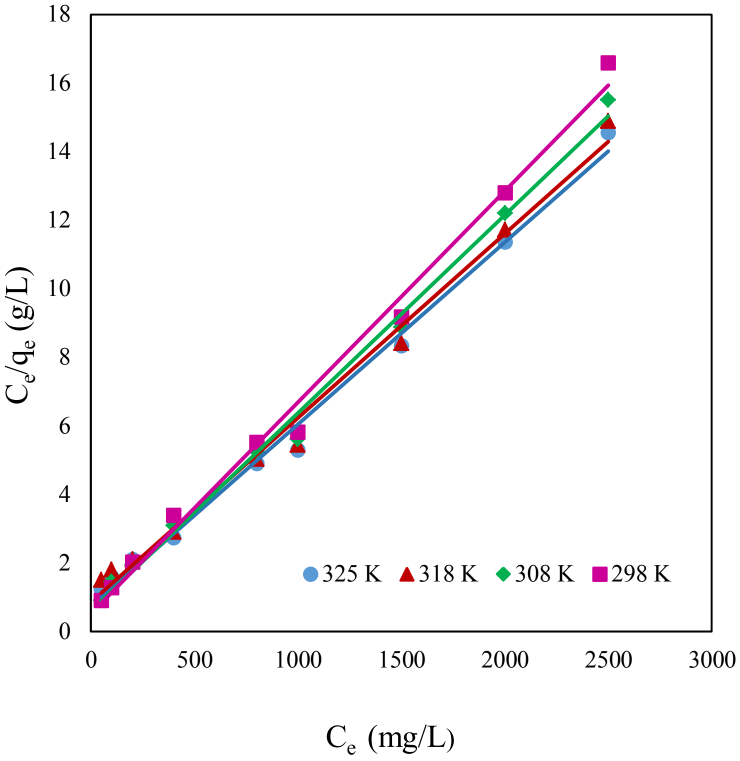

Fig. 6Sa. The linear plot of Langmuir adsorption isotherm

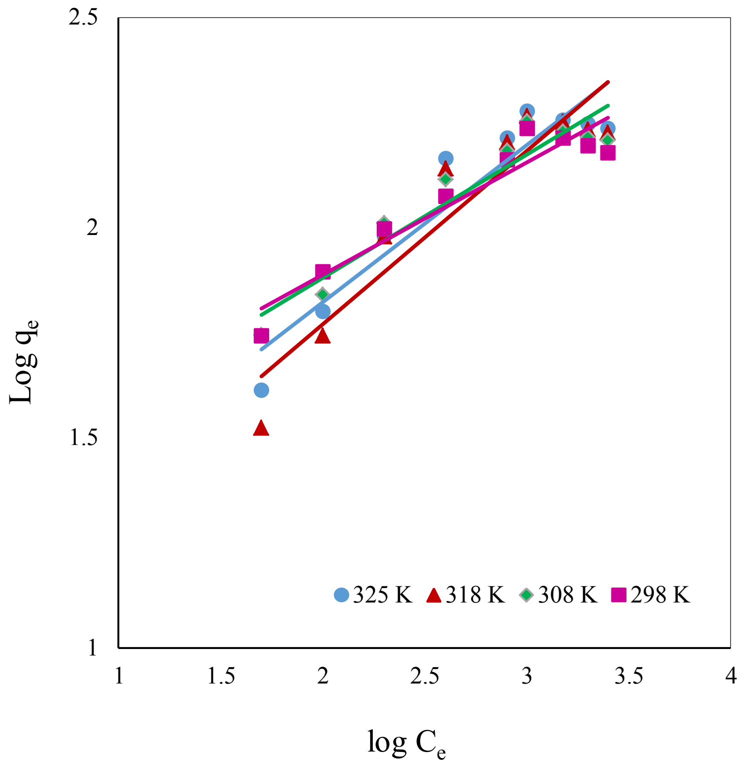

Fig. 6Sb. The linear plot of Freundlich adsorption isotherm

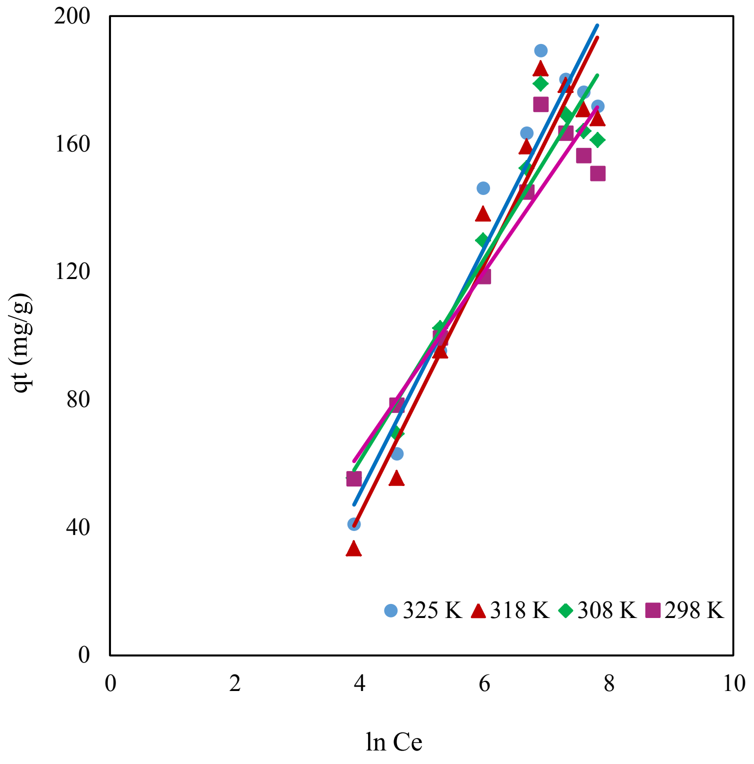

Fig. 6Sc. The linear plot of Temkin adsorption isotherm

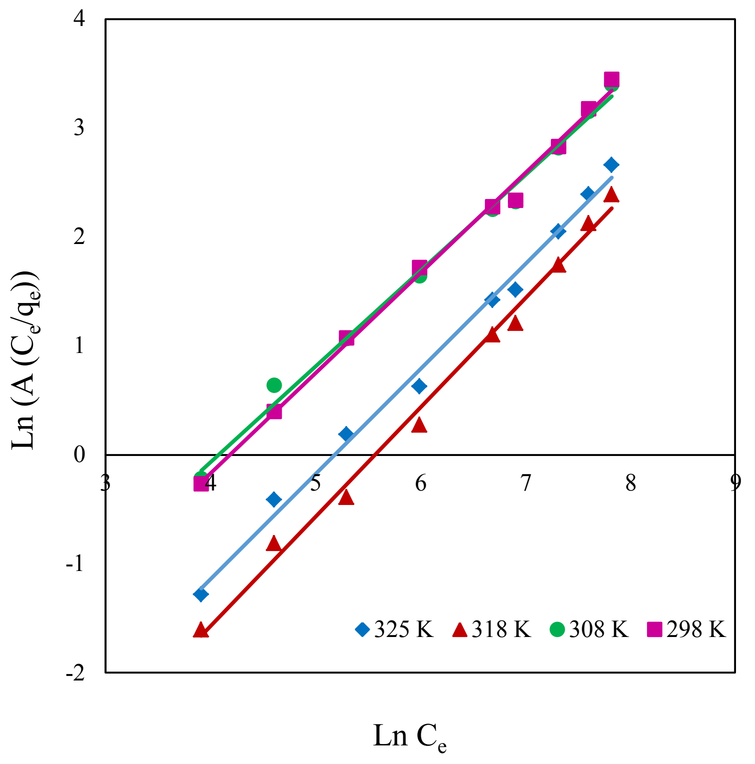

Fig. 6Sd. The linear plot of Redlich-Peterson adsorption isotherm

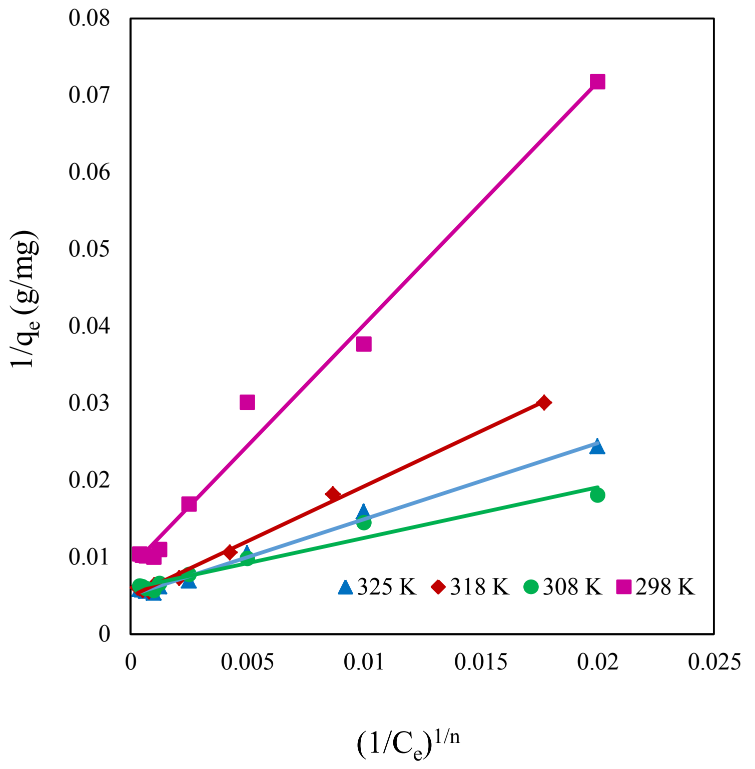

Fig. 6Se. The linear plot of Sips adsorption isotherm

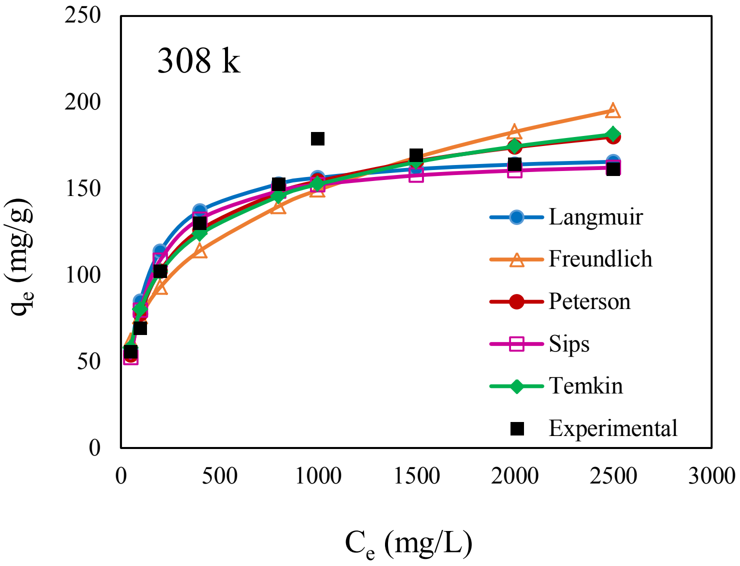

Fig. 7Sa. The non-linear plot of Langmuir, Freundlich, Temkin, Redlich-Peterson, Sip adsorption isotherms and experimental data at 308 K

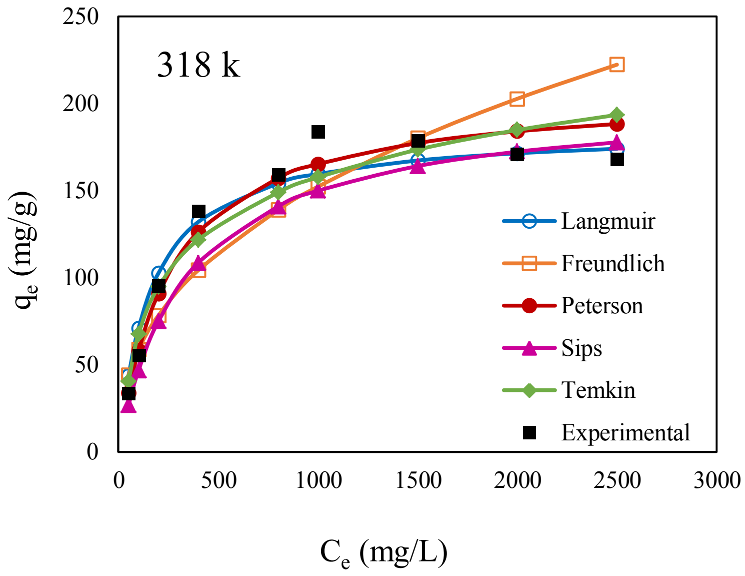

Fig. 7Sb. The non-linear plot of Langmuir, Freundlich, Temkin, Redlich-Peterson, Sip adsorption isotherms and experimental data at 318 K

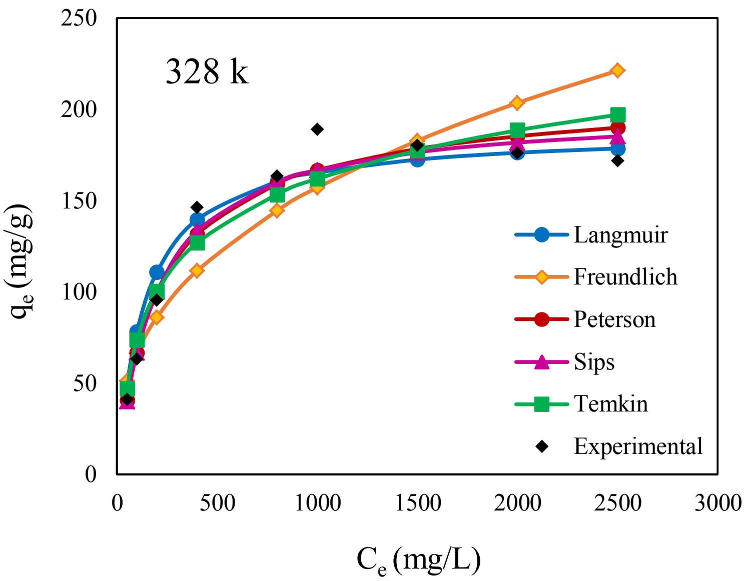

Fig. 7Sc. The non-linear plot of Langmuir, Freundlich, Temkin, Redlich-Peterson, Sip adsorption isotherms and experimental data at 328 K

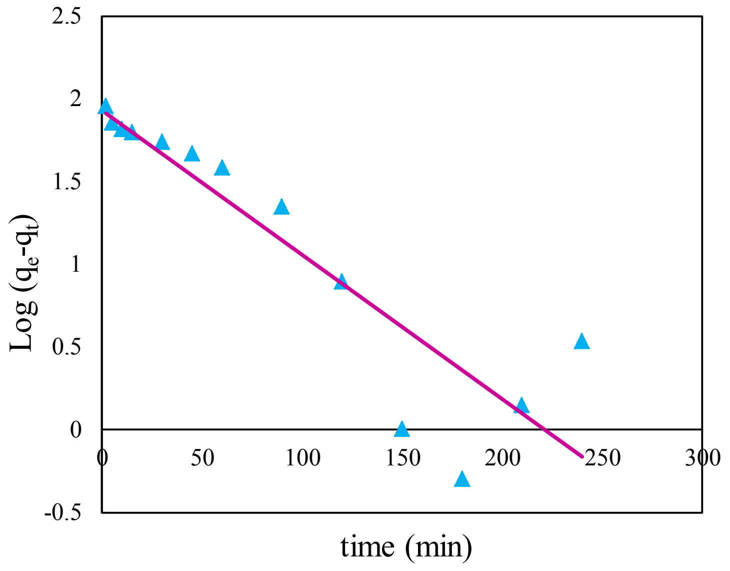

Fig. 8S. The linear plot of Pseudo-1st order kinetic model

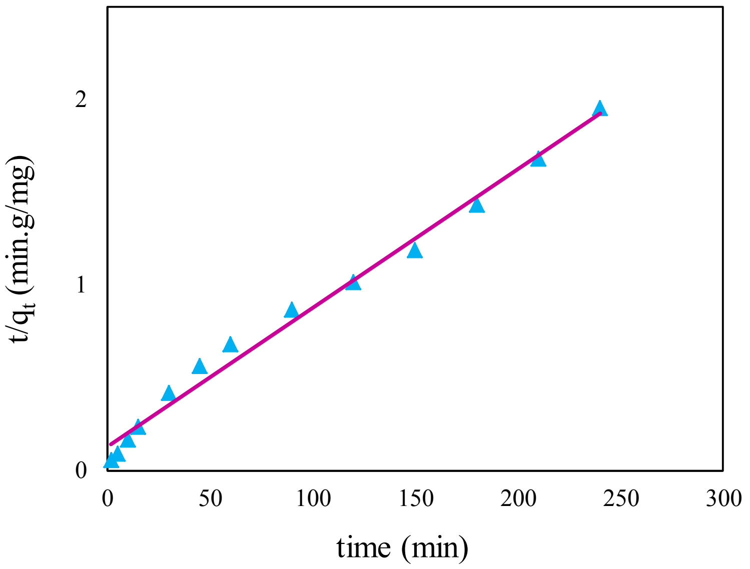

Fig. 9S. The linear plot of Pseudo-2nd order kinetic model

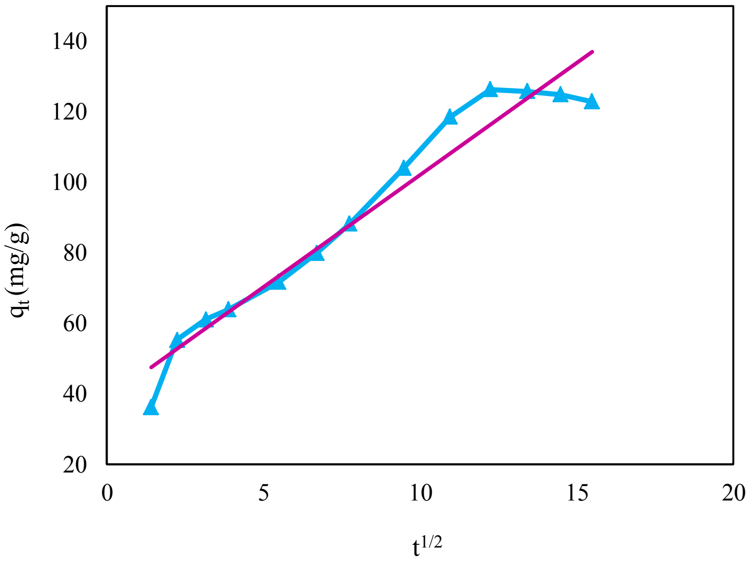

Fig. 10S. The linear plot of Intraparticle diffusion kinetic model

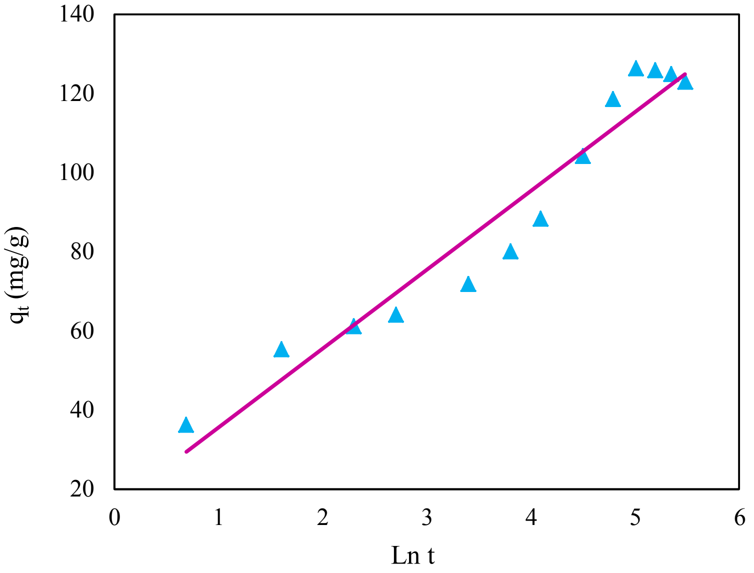

Fig. 11S. The linear plot of Elovich kinetic model

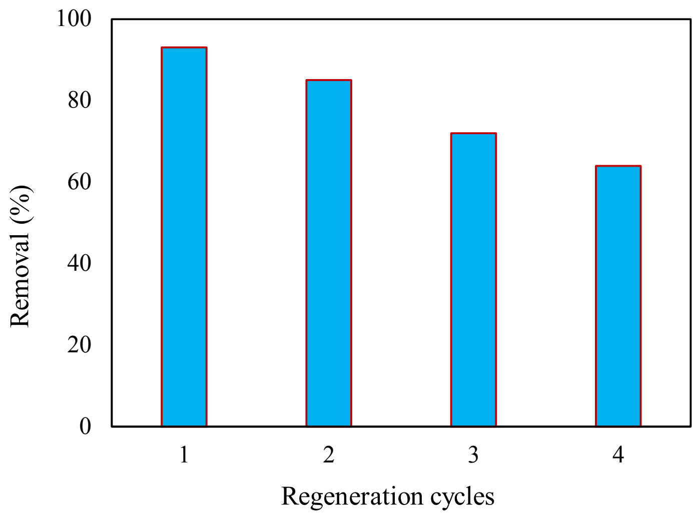

Fig. 12S. Reusability of the adsorbent

Table 1S. Thermodynamic parameters for Hg^{2+} adsorption on Fe_3O_4/PPy

Thermodynamic parameters						R^2
ΔH° (J/mol)	ΔS° (J/mol.K)	ΔG° (kJ/mol)				
		298 K	308 K	318 K	328 K	
0.24	52.69	-15.70	-16.23	-16.75	-17.28	0.9206

Table 2S. Langmuir, Freundlich and Temkin isotherms parameters and error analysis values

Isotherm	linear Expression	Parameters				
		**T(K)**	**298**	**308**	**318**	**328**
Langmuir	$\frac{C_e}{q_e} = \frac{1}{q_m K_L} + C_e \left(\frac{1}{q_m}\right)$	$\mathbf{K_L}$	0.3 E-2	9.7 E-2	6.2 E-2	7.1 E-2
		$\mathbf{q_m}$	114.95	172.41	185.18	188.68
		$\mathbf{R^2}$	0.9829	0.9946	0.9911	0.9928
		$\aleph^2$	6.95	8.17	12.54	11.77
		**ARE**	0.02	0.03	0.05	0.03
		**SSE**	47 E1	10 E2	11 E2	11 E2
Freundlich	$\log(q_e) = \log(K_F) + \frac{1}{n}\log(C_e)$	$\mathbf{K_F}$	2.34	0.25	8.78	11.87
		**n**	1.95	0.62	2.42	2.67
		$\mathbf{R^2}$	0.9296	0.9623	0.9431	0.8873
		$\aleph^2$	23.55	19.27	32.08	37.91
		**ARE**	0.3 E-2	0.6 E-2	1.7 E-2	1.4 E-2
		**SSE**	22 E2	29 E2	39 E2	58 E2
Temkin	$q_e = B_T \mathrm{Ln}\,(K_T) + B_T \mathrm{Ln}\,(C_e)$	$\mathbf{K_T}$	5.4 E-2	0.13	5.6 E-2	6.8 E-2
		$\mathbf{b_T}$	82.63	78.53	63.35	64.63
		$\mathbf{R^2}$	0.9304	0.9139	0.9164	0.911
		$\aleph^2$	170.19	9.64	15.63	14.78
		**ARE**	0.5	9.3 E-3	1.1 E-2	1.0 E-2
		**SSE**	95 E2	14 E2	20 E2	21 E2

Table 3S. Redlich-Peterson and Sips isotherms parameters and error analysis values

Isotherm	linear Expression	Parameters				
		**T(K)**	**298**	**308**	**318**	**328**
Redlich-Peterson	$\ln(A\frac{Ce}{qe} - 1) = g\ln(Ce) + \ln(B)$	**A**	0.34	2.00	0.80	1.00
		**B**	0.3 E-2	2.7 E-2	3.8 E-2	6.8 E-2
		**g**	0.89	0.88	1.00	0.96
		**R^2**	0.9117	0.9931	0.9932	0.9936
		$\aleph^2$	16.43	7.70	4.53	7.26
		**ARE**	0.42	5.7 E-2	1.6 E-2	6.6 E-3
		**SSE**	15 E2	11 E2	69 E1	11 E2
Sips	$\frac{1}{q_e} = \frac{1}{Q_{max}K_s}(\frac{1}{C_e})^{1/m} + (\frac{1}{Q_{max}})$	**K_S**	2.7 E-2	8.9 E-2	3.4 E-2	0.5 E-2
		**Q_{max}**	116.28	169.50	204.08	200
		**m**	1.00	1.00	0.96	1.00
		**R^2**	0.9975	0.9587	0.9962	0.9926
		$\aleph^2$	6.08	7.60	22.87	4.59
		**ARE**	1.0 E-2	0.01	0.14	4.3 E-3
		**SSE**	440.34	10 E2	31 E2	940.0

Table 4S. Kinetic parameters for Hg^{2+} adsorption onto Fe_3O_4/PPy

Kinetic model	Linear expression	Non-linear expression	parameters	
Pseudo first order	$\log(q_e - q_t) = \log q_e - (\frac{K_1}{2.303})t$	$q_t = q_e\,[1 - e^{-Kt}]$	q_e (mg/g)	85.27
			K_1 (1/min)	0.02
			R^2	0.81
			$\aleph^2$	247.92
			ARE	0.486
			SSE	19 E3
Pseudo second order	$\frac{t}{q_t} = \frac{1}{K_2 q_e^2} + \left(\frac{1}{q_e}\right)t$	$q_t = \frac{tK_2 q_e^2}{1 + tK_2 q_e}$	q_e (mg/g)	133.34
			K_2 (g/mg.min)	4.3 E-4
			R^2	0.98
			$\aleph^2$	37.69
			ARE	0.06
			SSE	21 E2
Elovich	$q_t = \frac{\ln(\alpha\beta)}{\beta} + \frac{1}{\beta}\ln(t)$	$q_t = \frac{1}{\beta}\ln(\alpha\beta t + 1)$	α (mg/g.min)	43.56
			β (g/mg)	0.05
			R^2	0.94
			$\aleph^2$	74.99
			ARE	0.20
			SSE	59 E2
Intraparticle diffusion	$q_t = K_i t^{\frac{1}{2}} + C_i$		K_i (mg/g.min$^{0.5}$)	6.34
			C_i (mg/g)	38.58
			R^2	0.94
